# Switchable MPC-based multi-objective regenerative brake control *via* flow regulation for electric vehicles

**DOI:** 10.3389/frobt.2023.1078253

**Published:** 2023-02-07

**Authors:** Mingming Mei, Shuo Cheng, Hongyuan Mu, Yuxuan Pei, Bo Li

**Affiliations:** ^1^ The State Key Laboratory of Automotive Safety and Energy, School of Vehicle and Mobility, Tsinghua University, Beijing, China; ^2^ The Institute of Industrial Science, University of Tokyo, Tokyo, Japan; ^3^ Beijing Automotive Industry Corporation, Beijing, China

**Keywords:** e-booster, switchable MPC, regenerative braking, flow regulation, pedal feel

## Abstract

Recent investigations of the electric braking booster (E-Booster) focus on its potential to enhance brake energy regeneration. A vehicle’s hydraulic system is composed of the E-Booster and electric stability control to control the master cylinder and wheel cylinders. This paper aims to address the independent closed-loop control of the position and pressure as well as the maintenance of the pedal feel. To track both the reference signals related to piston displacement and the wheel cylinder pressure, an explicit model predictive control (MPC) is developed. First, the new flow model is introduced as the foundation for controller design and simulation. Next, in accordance with the operational conditions, the entire system is divided into three switchable subsystems. The three distributed MPCs are constructed based on the linearized subsystems, and a state machine is used to perform the state jump across the controllers. A linear piecewise affine control law can then be obtained by solving the quadratic program (QP) of explicit MPC. Afterwards, the non-linear extended Kalman filter including the recorded time-variant process noise is used to estimate all the state variables. The effectiveness of the explicit MPC is evidenced by the simulations compared with a single MPC in regenerative and dead-zone conditions. The proposed controller decreases the latency significantly by 85 milliseconds, which also helps to improve accuracy by 22.6%. Furthermore, the pedal feel remains consistent, even when factoring in the number of vibrations caused by the inherent hydraulic characteristic of pressure versus volume.

## 1 Introduction

Electric vehicles (EV) have become a central focus of the automotive industry. The electric braking booster (E-Booster) system has been widely commercialized due to its compatibility with the existing hydraulic system and potential for enhancing energy harvesting efficiency. It can also directly manipulate the piston of the master cylinder in order to adjust the brake pressure. Existing methods of implementing the power-assisted brake can be broken up into two categories: one is where an emulator simulates the pedal feel, and the power assist is accomplished by means of the closed-loop of pressure or torque Liu et al. (2016); Yu et al. (2016); Yang et al. (2012); and the other category involves translating the force feedback problem to the position tracking control based on the reaction disk’s state of deformation Wu et al. (2020); Chen et al. (2018a), Chen et al. (2018b). With the latter category, which makes use of a non-contact electromagnetic transducer, there is higher accuracy, and the stroke sensor is more easily installed Wu et al. (2020). Additionally, due to the highly non-linear and time-varying relationship between the position and pressure Yu et al. (2016); Panzani et al. (2014), the pressure controller needs to overcome the challenging problem of the dead zone. In general, the position loop is regarded as a necessary control logic of the E-Booster.

The E-Booster expands the application field of the regenerative braking system (RBS), which lays the foundation for cooperative brake control. The force of the friction between the brake pad and the disk, as well as the regenerative force of the electric motor, mainly consists of the blending braking force. RBS normally prioritizes energy recuperation efficiency. Heydari et al. (2020) optimized the regenerative braking within the boundaries, utilizing a performance map of the traction motor. Furthermore, vehicle stability in braking situations needs to be taken into account. In Tang et al. (2022), an optimal balance method between braking safety and efficiency was developed. This method consisted of two components: one was a force distribution controller, and the other was an optimization object that was based on the wheel slip rate. A supervised learning technique was developed in the literature Changran et al. (2018) in order to achieve greater balance between both braking stability and efficiency. Given the difference in brake sources, the consistency of the braking feel has also been modified; Wang et al. (2018) presented a braking feel compensator to account for hydraulic hysteresis in the presence of reasonable braking force distributions. Here, the E-Booster plays an intricate role in maintaining a consistent pedal feel; this is due to its adjustable boosting ratio; Ohtani et al. (2011) designed the springs to act as a much-needed balance between the driver force and the motor force; Zhao et al. (2018) proposed a sliding mode controller (SMC) for regulating the motor torque and antilock braking system (ABS). This was shown to have the possibility to simultaneously adjust the pedal feel and wheel cylinder pressure. It was also mentioned in Zhao’s findings that the work of electric stability control (ESC) is separated into three stages based on the measured regenerative conditions Zhao et al. (2019). Considering the above-mentioned research, this paper will mainly highlight multi-objective optimization in the generative process using the E-Booster and hydraulic circuit as the actuation foundation.

The model predictive control (MPC) is a common method used for dealing with multi-target and multi-restriction problems. In Falcone’s study, a combined steering and braking MPC algorithm Falcone et al. (2008) was presented based on two models of varying complexity. The comprehensive controller was observed to have performed better under all operating conditions; however, it had a high computational burden. Meanwhile, the simplified controller behaved well under specific conditions. Taking the gathered data into consideration, this paper Cairano et al. (2013) proposed an MPC to coordinate the active front steering and differential braking. This method of predicting the key states using a piecewise affine (PWA) system provided the advantage of a low computation load. Hu et al. (2021) also introduced a gain-scheduled MPC to cover the entire range by a group of linearized models. This study will take a similar approach, with one exception: the linearization will be dependent on the control mode. MPC has naturally been discussed in relation to RBS. In his literature, Satzger and Castro (2014); Satzger et al. (2016); Satzger and de Castro (2017); Satzger and Castro (2018), proposed the MPC framework for tracking the desired torque by maximizing the regenerative efficiency and optimizing the slip ratio. Additionally, other control criteria, such as automotive yaw stability, were included into the MPC cost function Ren et al. (2016). To solve the problem of low computation efficiency, a nearest point (NP) algorithm was introduced to confront the increasingly complex objectives and constraints Li et al. (2016). The online solver of the quadratic program (QP) accounts for the large amount of computing resources. This is not feasible for automotive underlying actuators, considering their manufactured, highly responsive processors. The explicit MPC is a suitable method for application in such scenarios as vehicle dynamics Cairano et al. (2013); Di Cairano et al. (2010), idle speed control Cairano et al. (2012), traction control Tavernini et al. (2019), and so on. The explicit MPC can be implemented during the same step as a typical proportional integration (PI) to obtain a better effect; however, to do this, a significant number of linear control gains is required, the number of which depends largely on the MPC constraints Bemporad et al. (2011). When dimensions, horizon, and constraints are not excessive, then explicit MPC can produce acceptable results.

Generally speaking, the previous research on the E-Booster has seldom considered the quantitative flow control in the hydraulic system as a primary focus, including both E-Booster and ESC. Based on the flow control, this paper will present solutions for independent hydraulic braking adjustment while still maintaining a consistent pedal feel. First, the boost process of the E-Booster will be illustrated in terms of a mathematical model. The flow model for the traditional independent two-circuit hydraulic system will be established and simplified accordingly. The model will then be linearized according to the control mode, and a state machine will be used to rotate between the linearized subsystems. The main contribution of this work is to enable multi-objective tracking for complex non-linear coordinated brake systems *via* a distributed sub-MPC architecture. An off-line explicit MPC technique will also be adopted to solve the QP problem as a way to decrease the computing burden. As the master controller, this suggested technique can then be further applied to regeneration and dead zone control.

The rest of this paper is organized as follows: Section 2 presents the model of the electro-hydraulic system and formulates the problems that can arise. In Section 3, there is a discussion of the linearization strategy for the non-linear system, and the explicit MPC is developed. In Section 4, the effectiveness of the method is demonstrated by the simulation results in contrast to the single MPC. And finally, Section 5, the conclusion, provides a summary of the results.

## 2 Modelling

**FIGURE 1:**
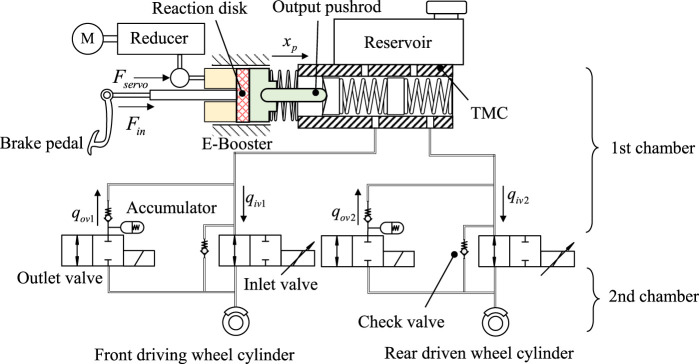
Simplified schematic diagram of the electro-hydraulic system.

The electro-hydraulic system with servo valves provided by ABS or ESC and powered by an E-Booster is the subject of this paper. Due to the symmetry of the brake circuit, one of the hydraulic circuits can be represented by the other in the H-type architecture. With unnecessary hydraulic components removed, the remaining simplified system contains a E-Booster, a tandem master cylinder (TMC) and two hydraulic circuits, which is depicted in Figure 1.

The hydraulic circuit is shaped like an H. Figure 1 illustrates the simplified arrangement. Each circuit is composed of an outlet valve, an inlet valve, a wheel cylinder, and a low-pressure accumulator. In Figure 1, *F* indicates the force applied to the reaction disk. Its subscripts indicate the source of the forces, whether it comes from the motor or the driver. *x*_*p*_ is the master cylinder piston displacement. *q* indicates the valve flow rate, and its subscripts are used to display whether it is an outlet valve or an inlet valve. The direction of the arrow next to these symbols indicates the direction where the value is positive.

### 2.1 The mechanical submodel

**FIGURE 2:**
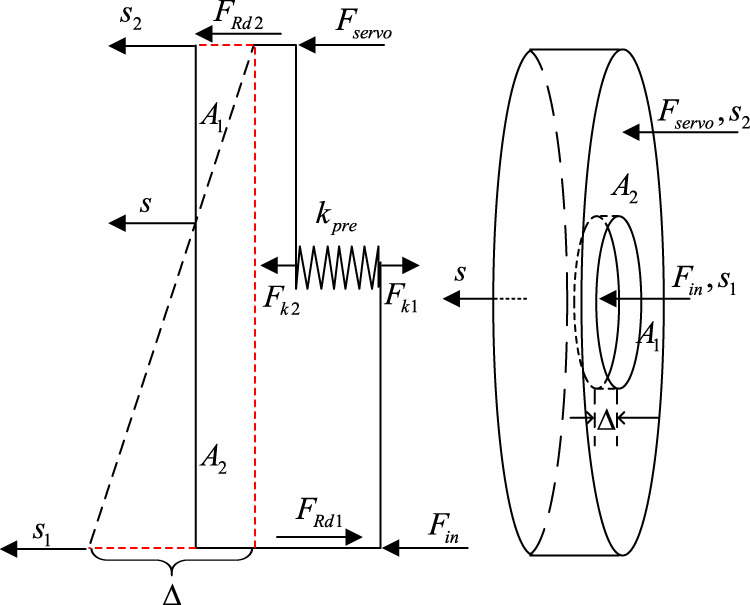
The diagram of the reaction disk.

The resultant force is obtained by coupling the input and the servo forces. Both forces act on the rubber-material reaction disk, the deformation of which influences the component forces’ interaction (shown in Figure 2).

The relationship between the input and servo forces can be written by the following equation

$$A_{1}\left(F_{\mathrm{servo}}+F_{\mathrm{Rd}2}+F_{k2}\right)-A_{2}\left(F_{in}-F_{\mathrm{Rd}1}-F_{k1}\right)=0$$ (1)

where *A*_1_, *A*_2_ are the cross-sectional area of the reaction disk’s center circle and outer ring. *F*_*servo*_ is the servo force provided by the motor. *F*_*in*_ is the force applied to the input rod by the driver. *F*_*Rd*1_, *F*_*Rd*2_ are the internal forces generated by the reaction disk’s deformation. *F*_*k*1_, *F*_*k*2_ are the spring force with the spring stiffness of *k*_*pre*_. *s*_1_, *s*_2_, *s* are the displacement of the center-circle surface, the concentric surface, and the other side of the reaction disk as shown in Figure 2. Δ is the subtraction result between *s*_1_ and *s*_2_ (Δ = *s*_1_−*s*_2_).

The dynamic equation of TMC’s first piston can be written as

$${\ddot{x}}_{p}=\frac{1}{m}\left(F_{\mathrm{servo}}+F_{in}-k_{mc}x_{p}-C{\dot{x}}_{p}-p_{m}A_{mc}\right)$$ (2)

where *x*_*p*_ is the displacement of piston and is equal to the above-mentioned *s*. *m* is the piston’s mass; *k*_*mc*_ is the equivalent stiffness including the return spring and the tandem springs in TMC chambers. *C* is the coefficient of friction force. *p*_*m*_ is the master cylinder pressure. *A*_*mc*_ is the TMC cross-sectional area.

### 2.2 The hydraulic submodel

With the inlet valve as the border, a simplified hydraulic circuit can be divided into two chambers (shown in Figure 1). The first chamber indicates the closed volume from the TMC to the inlet valve, while the second chamber represents the wheel cylinder. The principle of equal-flow exchange exists between the chambers. The inlet and outlet valves are the actuators for regulating the flow. The inlet valves model can be expressed by

$$q_{iv}=\left\{\begin{array}{cc} C_{q}\lambda_{iv}A_{iv}\sqrt{\frac{1}{\rho }\left|p_{m}-p_{w}\right|} & if\left(p_{m}-p_{w}\right)>-p_{\mathrm{crack}}\\ -C_{q}\lambda_{iv}\left(A_{iv}+A_{cv}\right)\sqrt{\frac{1}{\rho }\left|p_{m}-p_{w}\right|} & if\left(p_{m}-p_{w}\right)\leq -p_{\mathrm{crack}} \end{array}\right.$$ (3)

where *q*_*iv*_ is the flow rate passing the inlet valve and the direction from TMC to wheel cylinder is positive. *C*_*q*_ is the flow discharge coefficient. *λ*_*iv*_ is the orifice opening with a range from 0 to 1. *A*_*iv*_, *A*_*cv*_ is the orifice passage area of the inlet and check valve. *ρ* is the brake fluid density. *p*_*w*_ is the wheel cylinder pressure. *p*_*crack*_ is the cracking pressure of the check valve. And the outlet valve is modelled by

$$q_{ov}=C_{q}\lambda_{ov}A_{ov}\sqrt{\frac{1}{\rho }p_{w}}$$ (4)

where *q*_*ov*_ is the flow rate passing the outlet valve; *λ*_*ov*_ is the orifice opening of the outlet valve with the value from 0 or 1. *A*_*ov*_ is the orifice area of the outlet valve.

The hydraulic model of TMC can be constructed based on the inlet valve’s flow rate. Its mathematical expression is as follows:

$$\left\{\begin{array}{cc} {\dot{p}}_{m} & =\frac{E_{m}}{V_{m}-A_{mc}x_{p}}\left(A_{mc}{\dot{x}}_{p}-q_{iv}\right)\\ {\dot{p}}_{w} & =\frac{E_{w}}{V_{w}}\left(q_{iv}-q_{ov}\right) \end{array}\right.$$ (5)

where *V*_*m*_, *V*_*w*_ are the dead volume of the first and second chambers. *E*_*m*_ is the brake fluid bulk modulus. *E*_*w*_ is the equivalent bulk modulus of the wheel cylinder.

To facilitate the controller design, the mathematical model mentioned in this section discards some non-linear characteristics, including the following: i) the non-linear curve is replaced by a proportional straight line in the quasi-static increasing process; ii) there is a dead zone before the pressure is built, i.e., the stroke of master cylinder increases, but the pressure remains 0; iii) there is a hysteresis characteristic of the hydraulic system, i.e., the pressure of the piston in the same position on the forward stroke is greater than the pressure on the return stroke. As for the first item, as long as the linearization of the equiproportional ratio is applied appropriately, the effect on the actual control is relatively small. Meanwhile, the design of the pressure dead zone is mentioned later. Concerning the hydraulic hysteresis characteristics, the system stiffness in the return stroke is less than the forward stroke, which may cause the system to converge slowly in the return process.

### 2.3 Problem formulation

H-shaped vehicles are more convenient for single-axle braking force control than X-shaped vehicles. The front-drive and rear-drive principles are the same for vehicles with single-axle independent control. In this paper, the front-wheel-drive (FWD) is considered as a research case, and its results can be extrapolated to single-axle-drive vehicles.

Hence, define the state variables $x={\left[\begin{array}{ccccc} x_{p}\; & {\dot{x}}_{p}\; & F_{in}\; & p_{m}\; & \begin{array}{cc} p_{wf} & p_{wr} \end{array} \end{array}\right]}^{T}$ and the input vector $u={\left[\begin{array}{ccc} F_{\mathrm{servo}} & \begin{array}{cc} q_{\mathrm{iv}1} & q_{\mathrm{ov}1} \end{array} & \begin{array}{cc} q_{\mathrm{iv}2} & q_{\mathrm{ov}2} \end{array} \end{array}\right]}^{T}$ , the entire state space can be given by

$$\left\{\begin{array}{cc} {\dot{x}}_{1} & =x_{2}\\ {\dot{x}}_{2} & =\frac{1}{m}\left(-k_{mc}x_{1}-Cx_{2}+x_{3}-A_{mc}x_{4}+u_{1}\right)\\ {\dot{x}}_{3} & =g_{I}\left(x_{1},x_{2},u_{1}\right)\\ {\dot{x}}_{4} & =\frac{E_{m}}{V_{m}-A_{mc}x_{1}}\left(A_{mc}x_{2}-q_{\mathrm{iv}1}-q_{\mathrm{iv}2}\right)\\ {\dot{x}}_{5} & =\frac{E_{w}}{V_{w}}\left(q_{\mathrm{iv}1}-q_{\mathrm{ov}1}\right)\\ {\dot{x}}_{6} & =\frac{E_{w}}{V_{w}}\left(q_{\mathrm{iv}2}-q_{\mathrm{ov}2}\right) \end{array}\right.$$ (6)

where *g*_*I*_ represents the mapping from *x*_1_, *x*_2_ and *u*_1_ to *x*_3_, which is determined by the state of the reaction disk. *p*_*wf*_ and *p*_*wr*_ are the front and rear wheel cylinder pressure. *q*_*in*1_ and *q*_*ov*1_ are front inlet and outlet valve flow. *q*_*in*2_ and *q*_*ov*2_ are front inlet and outlet valve flow. the signs of flow variables are positive when the flow directions come from the TMC to wheel cylinders.

The pressure in the TMC and wheel cylinder are decoupled during regenerative braking, thus the pedal feel is not always consistent with normal braking. The relationship between piston displacement *x*_*p*_ and the driver’s input force *F*_*in*_ is used to define the pedal feel in this study.

A MPC is developed to realize the precise regulation of both *x*_*p*_ and *p*_*w*_, which indicates the decoupling of pedal feel and the wheel cylinder pressure. Another goal of the controller is to maintain brake-feel consistency during regenerative braking.

## 3 Controller design

### 3.1 Non-linear model linearization

The non-linear model needs to be transformed into a local linear time-invariant (LTI) model by linearization and local simplification as the foundation of MPC design. Under ideal circumstances, the input force *F*_*in*_ is proportional to the piston displacement *x*_*p*_. To ensure consistency of the pedal feel *x*_3_ can be written as follows:

$${\dot{x}}_{3}\approx \sigma {\dot{x}}_{2}$$ (7)

where *σ* is the ideal pedal-feel coefficient between the displacement and input force.

When 7) is introduced into 6), by calculating the Jacobian matrix at the nominal point, the non-linear system can be converted to a linear system as follows:

$$\dot{x}\left(t\right)-f\left(t_{0},x_{0},u_{0}\right)={\left.\frac{\partial f}{\partial x}\right|}_{t_{0},x_{0},u_{0}}\left(x\left(t\right)-x_{0}\right)+{\left.\frac{\partial f}{\partial x}\right|}_{t_{0},x_{0},u_{0}}\left(u\left(t\right)-u_{0}\right)$$ (8)

**FIGURE 3:**
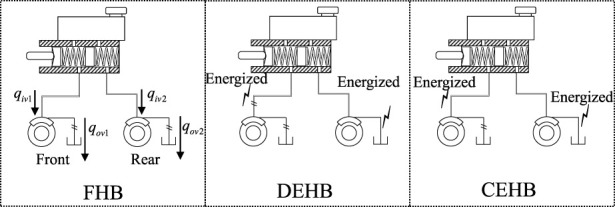
The simplified diagram of hydraulic work modes.

As shown in Figure 3, the hydraulic system is divided into three work modes during the regenerative braking process: fully hydraulic braking (FHB), distributed electro-hydraulic braking (DEHB), and coordinated electro-hydraulic braking (CEHB). The hydraulic system operates normally in FHB mode, with solenoid valves deactivated. The vehicle’s braking forces are all provided by the clamping force of the braking disc. When the motor’s regenerative braking is sufficient to meet the braking requirements of the driving wheels, the operating mode is switched to DEHB. The flow of the front wheels *q*_*iv1*_ is limited to zero, and the rear outlet valve is used to regulate the rear wheel cylinder pressure. Similarly, in the CEHB mode, the continuously adjustable *q*_*iv1*_ determines the front wheel cylinder pressure, allowing for blending braking of the driving wheels. It should be noted that the volume of a low-pressure accumulator is large enough that its internal pressure is almost zero.

**FIGURE 4:**
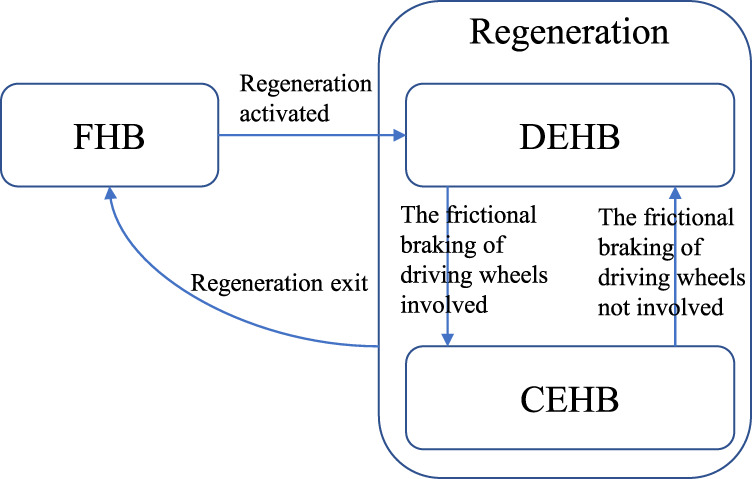
State machine for the control mode.

The whole LTV plant is classified into three LTI sub-models in terms of control patterns. The state transition matrix set is solved by Eq. 8 as $\left\{A_{k},B_{k},C_{k}\right\}\subseteq \left\{A_{j},B_{j},C_{j}\left|j\in \left\{1,2,3\right\}\right.\right\}$ , corresponding to FHB, DEHB, and CEHB in turn. The state at time *k* is manipulated by a state machine as shown in Figure 4.

**FIGURE 5:**
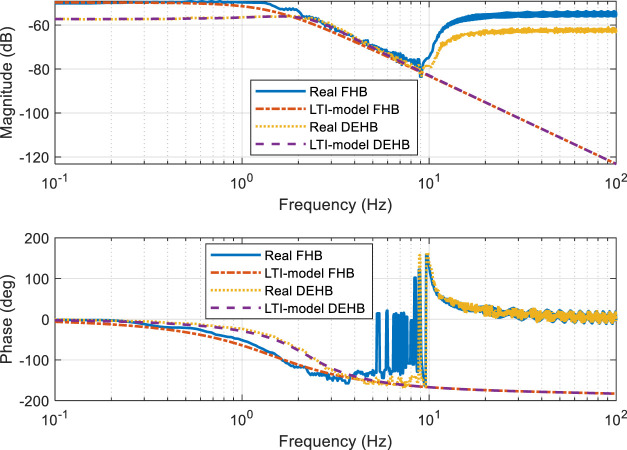
The Bode figure of TMC pressure vs. input force.

Figure 5 illustrates a comparison of the linearized and original models. The relationship between *x*_*p*_ and *F*_*servo*_ has changed while *q*_*iv*_ is set to 0. The amplitude-frequency curve is moved down, and the phase frequency is also changed. Both linearized sub-models are capable of fitting actual curves.

### 3.2 Switchable MPC design

The MPC controller is introduced to solve the non-linear problem. It’s a method for online optimization with a finite horizon. This is a multi-objective, multi-constraint optimization problem. MPC contains three features: model-based prediction, moving horizon optimization, and a framework that includes both feedforward and feedback. The non-linear electro-hydraulic model is simplified into the piecewise linear model in the previous section.

**FIGURE 6:**
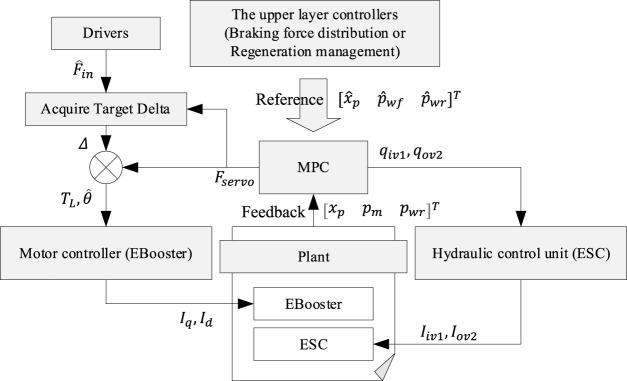
The framework of the MPC.

The controller’s objective is to implement multiple target tracking. The placement of MPC in the controller structure is shown in Figure 6. MPC receives the tacking reference from the upper-layer controllers, and then it’s the lower-layer actuators (EBooster and ESC) that conduct the commands from MPC. The control instructions are the increment signal to restrain the static error. The optimal objectives not only include the output references but also the input increments and input references. The increments decide whether MPC is conservative or aggressive. The input references can avoid the windup effect caused by the saturation of input and output.

The optimal constrained MPC problem can be formulated as follows:

$$\begin{array}{cc} \underset{z}{\min} & \begin{array}{c} \overset{N-1}{\underset{k=0}{\sum }}{\left\|W_{y}\left(y\left(t+k|t\right)-y_{\mathrm{ref}}\left(t\right)\right)\right\|}_{2}^{2}\\ \;+{\left\|W_{\Delta u}\Delta u\left(k\right)\right\|}_{2}^{2}+{\left\|W_{u}\left(u\left(t+k|t\right)-u_{\mathrm{ref}}\left(t\right)\right)\right\|}_{2}^{2} \end{array}\\ s.t. & \left\{\begin{array}{c} x\left(t+1\right)=A_{j}x\left(t\right)+B_{j}u\left(t\right)\\ y\left(t\right)=C_{j}x\left(t\right), \end{array}\right.j\in \left\{1,2,3\right\}\\ & \Delta u_{\min}\leq \Delta u\leq \Delta u_{\max}\\ & \Delta u_{k}=0,\;k=N_{u},\ldots ,N-1\\ & u_{\min}\leq u_{k}\leq u_{\max},\;k=0,\ldots ,N_{u}-1\\ & y_{\min}\leq y_{k}\leq y_{\max},\;k=1,\ldots ,N_{c} \end{array}$$ (9)

where *W*_*y*_, *W*_Δ*u*_, *W*_*u*_ are the diagonal tunning weight matrices for output signals and input increments. *N*, *N*_*u*_, *N*_*c*_ are the prediction horizon, the input horizon, and the constraints horizon, respectively.

According to the regenerative operating condition and control logic, a finite state machine is proposed to execute the state jump between the modes. The solution of MPC is presented in the single state where the state space can be regarded as the LTI in this following section.

Define $\bar{x}={\left[\begin{array}{cccc} x^{T} & y_{\mathrm{ref}}{\left(t\right)}^{T} & u{\left(t-1\right)}^{T} & u_{\mathrm{ref}}{\left(t\right)}^{T} \end{array}\right]}^{T}$ and $z={\left[\begin{array}{ccc} \Delta u_{0} & \cdots & \Delta u_{N-1} \end{array}\right]}^{T}$ , the input sequence can be expressed by the input increment

$$\left[\begin{array}{c} u_{0}\\ \vdots \\ u_{N-1} \end{array}\right]=\left[\begin{array}{ccc} I^{u} & & \\ \vdots & \ddots & \\ I^{u} & \cdots & I^{u} \end{array}\right]z+\left[\begin{array}{cccc} 0^{1\times x} & 0^{1\times y_{\mathrm{ref}}} & I^{1\times u} & 0^{1\times u_{\mathrm{ref}}}\\ \vdots & \vdots & \vdots & \vdots \\ 0^{1\times x} & 0^{1\times y_{\mathrm{ref}}} & I^{1\times u} & 0^{1\times u_{\mathrm{ref}}} \end{array}\right]{\bar{x}}_{0}$$ (10)

where the superscript denotes the dimension.

The output can be predicted by the state-space model as follows

$$\left[\begin{array}{c} y_{1}\\ y_{2}\\ \vdots \\ y_{N} \end{array}\right]=\left[\begin{array}{cccc} C & & & \\ CAB & C & \\ \vdots & \vdots & \ddots & \\ CA^{N-1}B & CA^{N-2}B & \cdots & C \end{array}\right]\left[\begin{array}{c} u_{0}\\ u_{1}\\ \vdots \\ u_{N-1} \end{array}\right]+\left[\begin{array}{c} CA\\ CA\\ \vdots \\ CA^{N} \end{array}\right]x_{0}$$ (11)

Then the simplification equations are obtained

$$\left[\begin{array}{c} y_{1}-y_{\mathrm{ref}}\\ \vdots \\ y_{N}-y_{\mathrm{ref}} \end{array}\right]=\Phi_{1}z+\Gamma_{1}{\bar{x}}_{0},\left[\begin{array}{c} u_{1}-u_{\mathrm{ref}}\\ \vdots \\ u_{N}-u_{\mathrm{ref}} \end{array}\right]=\Phi_{2}z+\Gamma_{2}{\bar{x}}_{0}$$ (12)

where Φ_1_, Φ_2_, Γ_1_, Γ_2_ are the constant matrices related to Eq. 10 and Eq. (11).

The weight matrices can be written by the weight vector

$${\bar{Q}}_{y}=blkdiag\left(\underset{N}{\underbrace{\begin{array}{ccc} {W_{y}}^{T}W_{y} & \cdots & {W_{y}}^{T}W_{y} \end{array}}}\right)$$ (13a)

$${\bar{Q}}_{\Delta u}=blkdiag\left(\underset{N}{\underbrace{\begin{array}{ccc} {W_{\Delta u}}^{T}W_{\Delta u} & \cdots & {W_{\Delta u}}^{T}W_{\Delta u} \end{array}}}\right)$$ (13b)

$${\bar{Q}}_{u}=blkdiag\left(\underset{N}{\underbrace{\begin{array}{ccc} {W_{u}}^{T}W_{u} & \cdots & {W_{u}}^{T}W_{u} \end{array}}}\right)$$ (13c)

The cost function is presented by

$$J\left(z,\bar{x}\left(t\right)\right)=\frac{1}{2}z^{T}Hz+\bar{x}{\left(t\right)}^{T}F^{T}z+\frac{1}{2}\bar{x}{\left(t\right)}^{T}Y\cdot {\bar{x}}_{0}$$ (14a)

$$H=2\left({\Phi_{1}}^{T}{\bar{Q}}_{y}\Phi_{1}+{\bar{Q}}_{\Delta u}+{\Phi_{2}}^{T}{\bar{Q}}_{u}\Phi_{2}\right)$$ (14b)

$$F^{T}=2\left({\Gamma_{1}}^{T}{\bar{Q}}_{y}\Phi_{1}+{\Gamma_{2}}^{T}{\bar{Q}}_{u}\Phi_{2}\right)$$ (14c)

$$Y=2\left({\Gamma_{1}}^{T}{\bar{Q}}_{y}\Gamma_{1}+{\Gamma_{2}}^{T}{\bar{Q}}_{u}\Gamma_{2}\right)$$ (14d)

When the constraints in Eq. 9 are transformed into the form of inequality, the standard form of QP is given by

$$\begin{array}{cc} \underset{z}{\min} & \frac{1}{2}z^{T}Hz+\bar{x}F^{T}z\\ s.t. & Gz\leq W+S\cdot \bar{x} \end{array}$$ (15)

This paper adopts an offline method to solve the QP. When the multi-parametric QP is from the MPC problem, the following matrix inequality is always satisfied Bemporad et al. (2002).

$$\left[\begin{array}{cc} Y & F^{T}\\ F & H \end{array}\right]\geq 0,H>0$$ (16)

The optimal *z* is continuous and PWA linear functions of *x* within the critical region Bemporad et al. (2002). Based on the non-negative least squares (NNLS) solver Bemporad (2015), the local control law can be described by the PWA function.

$$\begin{array}{cc} \Delta u^{*}\left(t\right)= & {\bar{F}}_{j}\bar{x}\left(t\right)+{\bar{G}}_{j}\\ & \;j:{\bar{H}}_{j}\bar{x}\left(t\right)\leq {\bar{K}}_{j} \end{array}$$ (17)

where $\bar{F}$ , $\bar{G}$ , $\bar{H}$ , $\bar{K}$ is the approximate multiparametric solutions.

**FIGURE 7:**
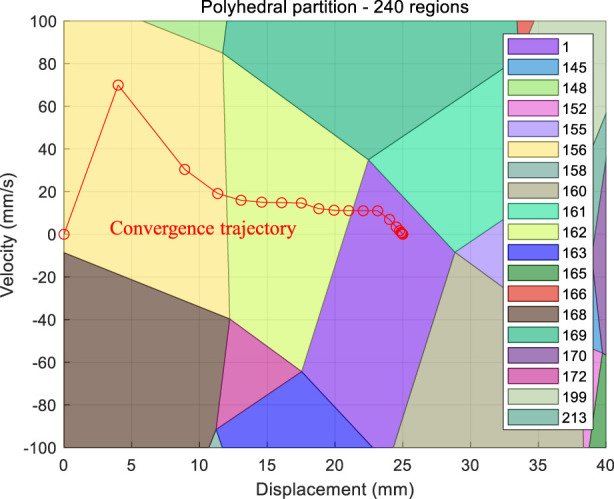
Polyhedral projection and convergence trajectory.

Mode CEHB is used as an example to draw a 2-dimensional projection of the polyhedral partition in the TMC piston displacement and piston velocity. The target displacement is 25 mm, and the remaining parameters without projection are set to 0. As shown in Figure 7, the polyhedra are divided into 240 blocks, and the actual trajectory converges from the initial point (0, 0) to the point (25, 0), *via* blocks 156 and 162 to block 1. This example shows that it is feasible to solve the QP problem by dividing the polyhedra offline.

### 3.3 State prediction

The proposed MPC needs all states as the feedback signals. If a linear Kalman filter is used, due to the non-linear characteristics of hydraulic systems, the process noise produces cumulative errors with the dynamic process. It will lead to significant deviations in the observed values and bring disturbances to the MPC. If the disturbance is too large, the controller may not converge. In order to eliminate cumulative errors, the state-space matrice needs to be updated in real time. As a result, an extended Kalman filter (EKF) is adopted to estimate states in the non-linear transition process. Since the linearization has been mentioned in the previous section, it’s not described in this section. The extended recursive equations of EKF is as follows

$$\hat{X}\left(k+1|k\right)=A_{k}X\left(k|k\right)+B_{k}u\left(k\right)$$ (18a)

$$P\left(k+1|k\right)=A_{k}P\left(k|k\right){A_{k}}^{T}+Q_{k}$$ (18b)

$$K\left(k+1\right)=P\left(k+1|k\right){C_{k}}^{T}{\left(C_{k}P\left(k+1|k\right){C_{k}}^{T}+R\right)}^{-1}$$ (18c)

$$\hat{X}\left(k+1|k+1\right)=\hat{X}\left(k+1|k\right)+K\left(k+1\right)\left(y\left(k\right)-C_{k}\hat{X}\left(k+1|k\right)\right)$$ (18d)

$$P\left(k+1\right)=\left(I-K\left(k+1\right)C_{k}\right)P\left(k+1|k\right)$$ (18e)

where *A*_*k*_, *B*_*k*_, *C*_*k*_ are the value of Jacobian matrix for the non-linear transition process at the time *k*.

The measurement noise covariance matrix *R* is time-invariant matrix. There is a large difference in the process noise of different so that the process noise covariance is time-varying. This paper proposes an on-line approach for the process noise, which includes both the priori tunning value and Sage-Husa maximum posterior estimation algorithm Myers and Tapley (1976).

Once the regenerative mode occurs change, the process noise ${\hat{Q}}_{t}$ is given the initial value ${Q_{0}}^{\left(i\right)}$ where *i* denotes the work mode at time *t*. During the same operating mode, ${\hat{Q}}_{t}$ can be updated by

$$d_{k}=\frac{1-b}{1-b^{k+1}}$$ (19a)

$${\hat{q}}_{k}=\left(1-d_{k-1}\right){\hat{q}}_{k-1}+d_{k-1}\left[{\hat{x}}_{k|k}-\left(A_{k}{\hat{x}}_{k|k-1}+B_{k}u_{k-1}\right)\right]$$ (19b)

$${\hat{Q}}_{k}=\left(1-d_{k-1}\right){\hat{Q}}_{k-1}+d_{k-1}\left[K_{k}\varepsilon_{k}{\varepsilon_{k}}^{T}{K_{k}}^{T}+P_{k}-A_{k}P_{k-1}A_{k}\right]$$ (19c)

where *k* is the period number from time to the current. *b* is the forgetting factor and *b* ∈ (.95, 1). *K*_*k*_ is the current Kalman gain. *ɛ*_*k*_ is the residual as follows

$$\varepsilon_{k}=y_{k}-C_{k}{\hat{x}}_{k|k-1}$$ (20)

### 3.4 The strategy for keeping pedal feel consistent

**FIGURE 8:**
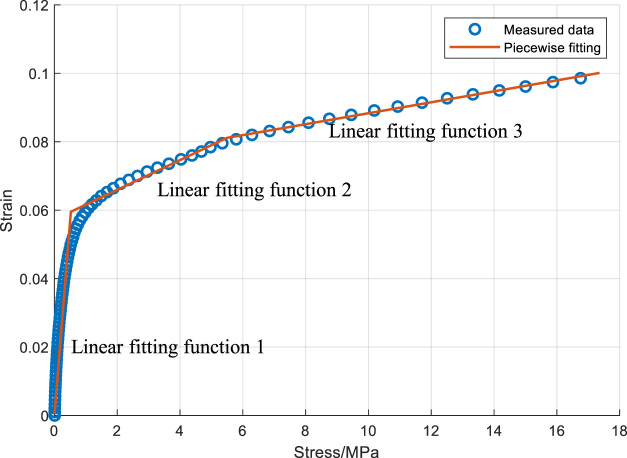
The stress vs. strain of the reaction disk.

Rubber material can be described by a non-linear GMM Jrad et al. (2012), which is divided into a quasi-static process and a dynamic process. Without the disturbance of rubber’s resilience, the static stressing and strain of rubber is shown in Figure 8. It’s the increasing curve with a decreasing slope, which can be fitted by a continuous three-piece linear function. By the means of the non-linear Trust-Region-Reflective Least Squares Moré and Sorensen (1983), the fitting piecewise function is obtained with the *R* ^2^ of .9700. It includes 5 parameters with 2 x-coordinates and 3 slopes. The detailed solution process is not discussed in this paper.

**FIGURE 9:**
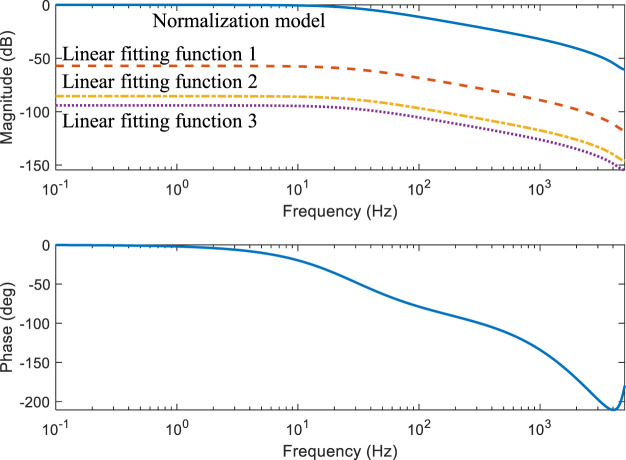
The Bode figure of reaction disk model.

The rubber’s dynamic characteristic is modelled by the Generalized Maxwell Model (GMM). A second-order transfer function with a zero point can be used to fit the reaction disk according to experimental data. Combined with the 3-piece linear fitting function, the frequency domain is presented in Figure 9. If the frequency is less than 10 Hz, the amplitude and phase can be regarded constant. Without considering the delay of controller, the phase frequency characteristics of the reaction disk reflect the hysteresis of the motor output force relative to the driver input force. When above 10 Hz frequency, the rubber hysteresis will exceed 20^◦^. It means that with the higher frequency of the driver input force, the motor output force will obtain the greater delay. The gain of amplitude-frequency curves in different pieces are proportional and the phase-frequency characteristics are identical.

According to the fitting second-order model, the deformation difference Δ (introduced in Eq. 1) can be calculated by

$$\begin{array}{cc} \left[\begin{array}{c} {\dot{\xi }}_{1}\\ {\dot{\xi }}_{2} \end{array}\right] & =\left[\begin{array}{cc} A_{Rd} & \\ & A_{Rd} \end{array}\right]\left[\begin{array}{c} \xi_{1}\\ \xi_{2} \end{array}\right]+\left[\begin{array}{cc} B_{\mathrm{Rd}1} & \\ & B_{\mathrm{Rd}2} \end{array}\right]\left[\begin{array}{c} F_{in}\\ F_{\mathrm{servo}} \end{array}\right]\\ \Delta & =\left[\begin{array}{cc} C_{\mathrm{Rd}1} & -C_{\mathrm{Rd}2} \end{array}\right]\left[\begin{array}{c} \xi_{1}\\ \xi_{2} \end{array}\right] \end{array}$$ (21)

where *ξ* is the state of the second-order model; *A*_*Rd*_ and *B*_*Rd*_ are time-invariant; *C*_*Rd*_ is the function of input force and $C_{Rd}\in \left\{C_{Rd}^{\mathrm{piece}1},C_{Rd}^{\mathrm{piece}2},C_{Rd}^{\mathrm{piece}3}\right\}$ .

The deformation difference can be measured by the displacement sensor. Its target value is acquired by the incremental updating method as

$$\dot{\Delta }=\left[\begin{array}{cc} C_{\mathrm{Rd1}}A & -C_{\mathrm{Rd}2} \end{array}A\right]\left[\begin{array}{c} \xi_{1}\\ \xi_{2} \end{array}\right]+\left[\begin{array}{cc} C_{\mathrm{Rd}1}B_{\mathrm{Rd}1} & -C_{\mathrm{Rd}2} \end{array}B_{\mathrm{Rd}2}\right]\left[\begin{array}{c} F_{in}\\ F_{\mathrm{servo}} \end{array}\right]$$ (22)

### 3.5 Dead zone

The proposed MPC method can be used to solve another engineering problem. Before the pressure building are two non-linear processes: idle and dead zone [7]. Idle denotes that the mechanical part is not connected to the hydraulic part, while they are connected in the dead zone.

The non-linear process can be regarded as a specific condition of mode DEHB. The idle and dead zone can be converted to the rear wheels’ virtual flow, and the reference outlet valve flow is equivalent to the virtual flow simultaneously. The controller for the idle and dead zone is given by

$$\begin{array}{c} \left\{\begin{array}{c} q_{\mathrm{vir}}=A_{mc}{\dot{x}}_{p}\\ {\hat{u}}_{3}=q_{\mathrm{vir}}\\ mpc:\left(10\right) \end{array}\right.\\ s.t.j=2\\ 0\leq x_{p}\leq x_{\mathrm{Idel}}+x_{\mathrm{Dead}} \end{array}$$ (23)

where *q*_*vir*_ is the virtual flow of rear wheels; ${\hat{u}}_{3}$ is the reference flow of rear outlet valves; *x*_*Idel*_, *x*_*Dead*_ are the idle travel and dead zone displacement.

## 4 Simulation results

Our entire model is composed of a series of local LTI models that are based on the proposed linearized method. The controller is designed based on the three LTI-MPCs, which correspond to the three regenerative conditions, which are FHB, DEHB, and CEHB. This explicit method is adopted for engineering applications, and an EKF observer is used to estimate all states with the assistance of the motor angle sensor and TMC pressure sensor. The performance of the controller is presented in contrast to that of a sub-MPC for CEHB, given that the control group is the only one capable of multi-objective optimization.

During the condition of FHB, electro-hydraulic solenoid valves are not activated to regulate the flow, and the E-Booster is the only actuator available for operating the pressure. The flow rates *q*_*ov1*_ and *q*_*ov2*_ are set to a value of zero resulting from the normally closed outlet valves. Hence, the force *F*_*servo*_ is the only applicable parameter, and the remaining *q*_*iv1*_ and *q*_*iv2*_ variables can be observed by using the Bernoulli equation.

**FIGURE 10:**
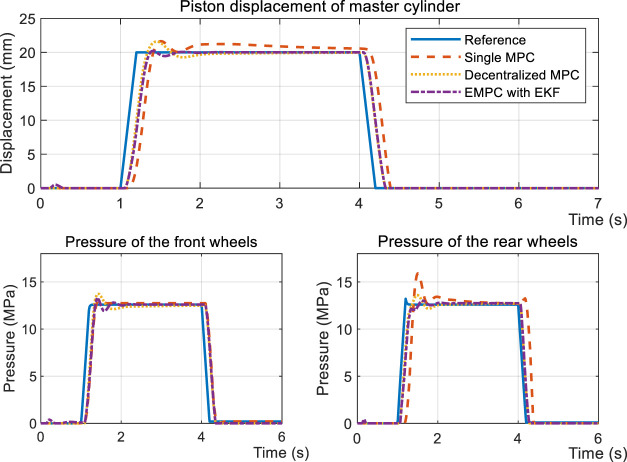
Simulation results of the step response during the FHB condition.

**FIGURE 11:**
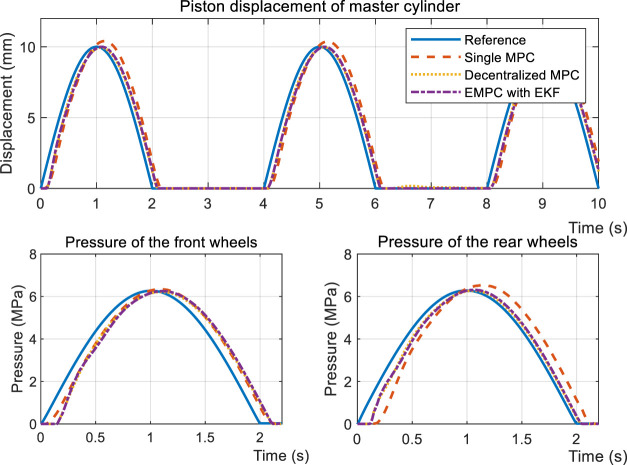
Simulation results of sinusoidal tracking under the FHB condition.

Figure 10 and Figure 11 both illustrate the controllers’ behaviors under the FHB condition. The step response and sinusoidal reference signals can be tracked by the controllers. The difference is that EMPC and DMPC (decentralized MPC) have improved the convergence rates in terms of piston displacement *x*_*p*_, given that there is an 85 ms shorter latency than the SMPC (single MPC). The overshoot of EMPC is greater than DMPC by 4.6% and SMPC by 22.6% due of the EKF observer.

**FIGURE 12:**
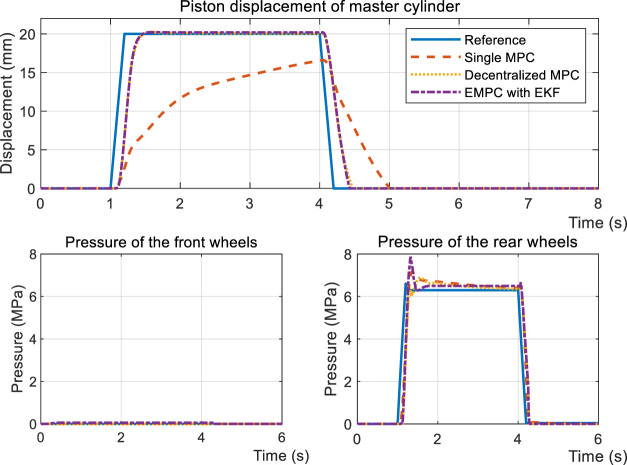
Simulation results of step response under the DEHB condition.

**FIGURE 13:**
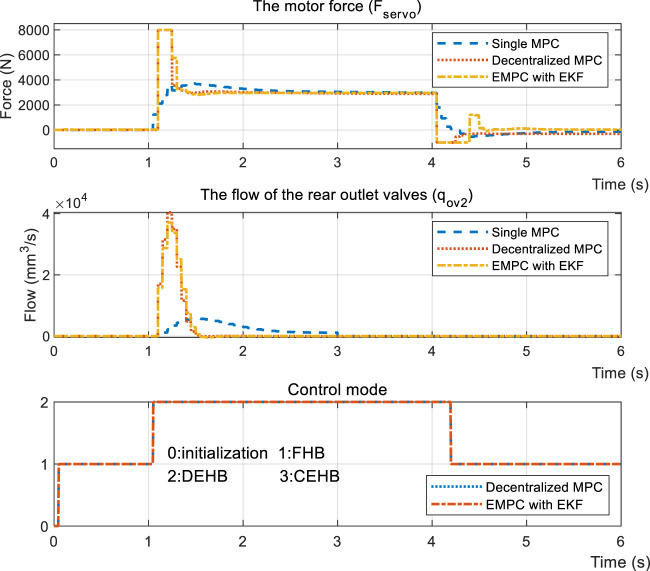
Input signal of step response under the DEHB condition.

Thanks to regeneration, part of the braking force is distributed to the driving motor. The vehicle type that is proposed is FWD. Therefore, it’s further subdivided into DEHB and CEHB, dependent on whether the front wheels have hydraulic braking force. Figure 12 depicts the dynamic performances when the desired front cylinder pressure is 0 and the rear cylinder pressure is half of the standard measure, compared with the same reference displacement *x*_*p*_. As shown in the figure, even though the pressure in the wheel cylinders is able to keep up with the reference, the displacement of SMPC still lags behind the objective curve. This results in the driver getting negative feedback from the pedal during regenerative braking. The input signal (Figure 13) further indicates that the conservative control strategy of the SMPC is insufficient for covering the entire regenerative condition.

**FIGURE 14:**
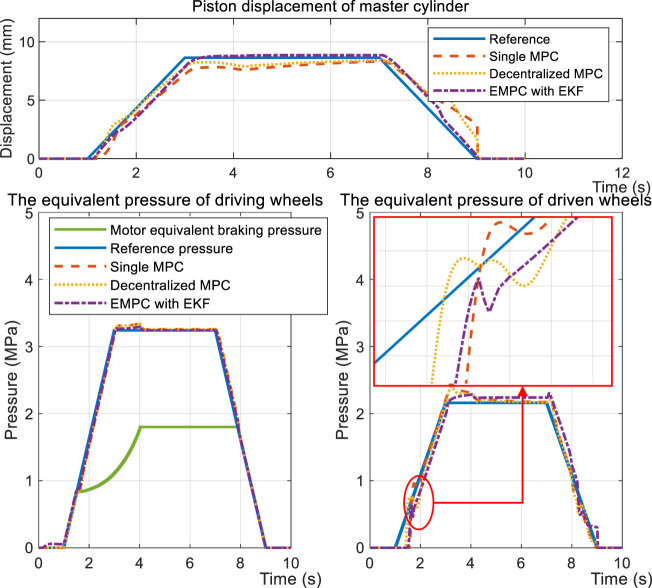
Regenerative simulation results of the test case.

**FIGURE 15:**
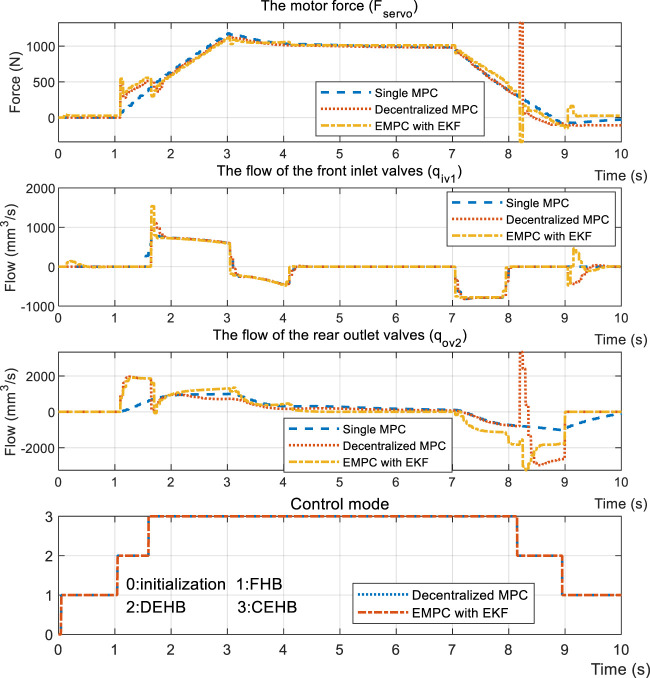
Input signal of the test case.

A normal regenerative example is provided to verify the overall performance of EMPC. After reaching an initial speed of 80 km/h, the vehicle then brakes at a gradually rising rate until reaching the maximum deceleration. The front braking force is shown to be proportional to the rear braking force in a constant 3:2 ratio. The constant power and torque areas are then traversed by the regenerative motor. It was observed that the performance of the EMPC is much better in terms of targets tracking, as highlighted in Figure 14. The main advantage here is the piston position control, which reaches a precision of ±.20 mm, compared with the ±.32 mm of DMPC and ±.45 mm of SMPC. The controllers display the same accuracy when it comes to driving wheels pressure; nevertheless, EMPC undergoes smaller fluctuations when the control mode is switched in relation to the driven wheel’s pressure regulation. The input signal is presented in Figure 15, where the inlet valve flow *q*_*iv1*_ is viewable as the net flow of all driving wheels. The negative difference can be counteracted by controlling *q*_*ov1*_ as shown in Figure 3.

**FIGURE 16:**
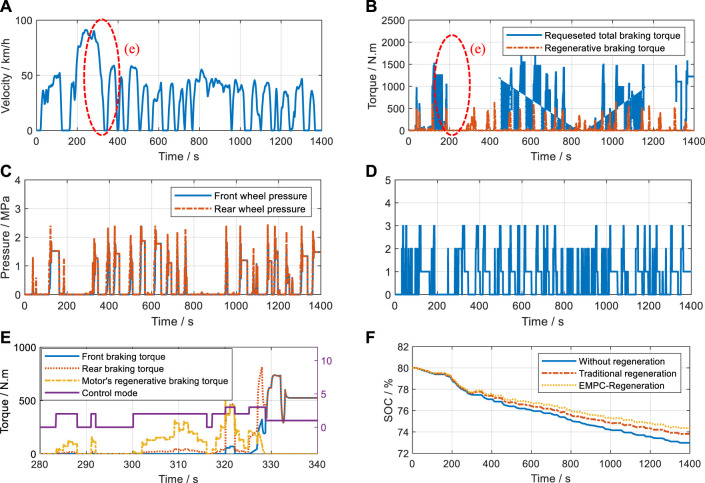
UDDS regeneration. **(A)** UDDS case. **(B)** The torque acting on the wheels. **(C)** Braking pressure. **(D)** Control mode. **(E)** The larger version of braking torque. **(F)** State-ofcharge.

The EMPC is applied in the UDDS (urban dynamometer driving schedule) case to demonstrate its effect on energy conservation. As seen in Figure 16, the sub-figure a) is the vehicle’s velocity in a cycle, and sub-figures (b)-(d) are the allotted regenerative and frictional braking torques in different control modes. The regenerative braking first intervenes, and the rear frictional braking then takes effect to guarantee longitudinal stability. Once the front and rear braking distribution exceeds the safety threshold the SMPC operates the front hydraulic pressure. Of all the braking length of time, 73.9% is in FHB, 17.6% in DEHB and 8.5% in CEHB. Without coordinated braking, the traditional regeneration separates the motor braking and hydraulic braking for the pedal feel and the driving safety. In terms of the proposed simulated model, the traditional regeneration saves 9.8% electric energy compared with the braking process without regeneration. While the switchable EMPC method can decrease 17.5% power consumption and improve 7.7% of regenerative efficiency.

**FIGURE 17:**
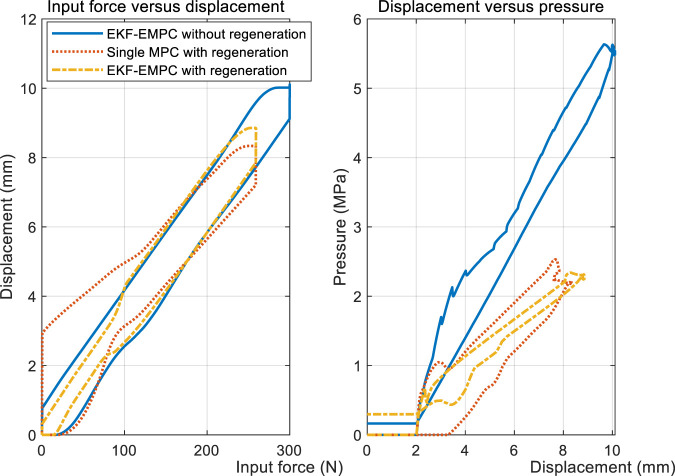
Pedal feel during regenerative braking.

One of the control targets is the consistence of driver’s pedal feel. The pedal feel is directely determined by the input force *F*_*in*_, which can be simplified to a variable proportional to *x*_*p*_ if the reaction disk is followed by the control law given in Eq. 22. Figure 17) shows the relationship between pedal force, pushrod displacement and TMC pressure, which directly reflects the hydraulic characteristics of the E-Booster and driver brake pedal feel. The control group of EMPC contains a normal braking case without regeneration, and a single MPC designed for CEHB only. Taking the pedal feel without regenerative braking as the control target, it can be seen from the left subfigure that both EMPC and single MPC grow smoothly at a slope of roughly .0335 mm/N during pressure increase. The single MPC shows a distinct hysteresis below 150 N during the pressure decrease process, while the EMPC can conform to the normal decompression pedal feel relatively well. As can be seen from the right subfigure, the slope of the p-V characteristic of the system in the regenerative braking case is .36 MPa/mm, which is only 53% of that in the case without regeneration. The combination of the two figures can show that: first, the pV characteristic changes significantly during regenerative braking, and a part of the hydraulic braking force is compensated by motor braking; second, the EMPC designed for the integrated three cases (as shown in Figure 3) can achieve the same braking feel as that without regeneration, and the effect is better than the single MPC, especially the decompression process.

## 5 Conclusion

A novel MPC-based method for pressure regulation of wheel cylinders during regeneration was developed. Because the desired values of displacement and pressure can be accurately tracked, we can guarantee a consistent pedal feel as indicated by the curve of the input force versus the displacement curve. The case comparison validates the effectiveness in all regenerative operating conditions. Using this method, not only can braking force be evenly distributed between the front and rear wheels, but the coordinated control of hydraulic electric braking in the driving wheels can also be realized. Furthermore, the method works effectively in hydraulic systems with different dead zones.

Future case studies will consider the effects of low-pressure accumulator volume and lateral motion to improve the accuracy of the results. They should also concentrate on improving the accuracy of flow control in consideration of additional factors. During future controller improvement, the disturbance caused by the hydraulic hysteresis will be paid more attention to accelerate the convergence speed of the return process.

## Data Availability

The raw data supporting the conclusion of this article will be made available by the authors, without undue reservation.
